# Complete genome sequence of the moderate thermophile *Anaerobaculum mobile* type strain (NGA^T^)

**DOI:** 10.4056/sigs.3547050

**Published:** 2013-04-15

**Authors:** Konstantinos Mavromatis, Erko Stackebrandt, Brittany Held, Alla Lapidus, Matt Nolan, Susan Lucas, Nancy Hammon, Shweta Deshpande, Jan-Fang Cheng, Roxanne Tapia, Lynne A. Goodwin, Sam Pitluck, Konstantinos Liolios, Ioanna Pagani, Natalia Ivanova, Natalia Mikhailova, Marcel Huntemann, Amrita Pati, Amy Chen, Krishna Palaniappan, Miriam Land, Manfred Rohde, Stefan Spring, Markus Göker, Tanja Woyke, John C. Detter, James Bristow, Jonathan A. Eisen, Victor Markowitz, Philip Hugenholtz, Hans-Peter Klenk, Nikos C. Kyrpides

**Affiliations:** 1 DOE Joint Genome Institute, Walnut Creek, California, USA; 2 Leibniz-Institute DSMZ - German Collection of Microorganisms and Cell Cultures, Braunschweig, Germany; 3 Los Alamos National Laboratory, Bioscience Division, Los Alamos, New Mexico, USA; 4 Biological Data Management and Technology Center, Lawrence Berkeley National Laboratory, Berkeley, California, USA; 5 Oak Ridge National Laboratory, Oak Ridge, Tennessee, USA; 6 HZI – Helmholtz Centre for Infection Research, Braunschweig, Germany; 7 University of California Davis Genome Center, Davis, California, USA; 8 Australian Centre for Ecogenomics, School of Chemistry and Molecular Biosciences, The University of Queensland, Brisbane, Australia

**Keywords:** Gram-negative, rod-shaped, motile, flagellum, non-spore forming, anaerobic, chemoorganotrophic, crotonate-reducer, *Synergistetes*, *Synergistaceae*, GEBA

## Abstract

*Anaerobaculum mobile* Menes and Muxí 2002 is one of three described species of the genus *Anaerobaculum*, family *Synergistaceae*, phylum *Synergistetes*. This anaerobic and motile bacterium ferments a range of carbohydrates and mono- and dicarboxylic acids with acetate, hydrogen and CO_2_ as end products. *A. mobile* NGA^T^ is the first member of the genus *Anaerobaculum* and the sixth member of the phylum *Synergistetes* with a completely sequenced genome. Here we describe the features of this bacterium, together with the complete genome sequence, and annotation. The 2,160,700 bp long single replicon genome with its 2,053 protein-coding and 56 RNA genes is part of the *** G****enomic* ***E****ncyclopedia of* ***Bacteria**** and* ***Archaea***** project.

## Introduction

Strain NGA^T^ (= DSM 13181 = ATCC BAA-54 = JCM 12221) is the type strain of *Anaerobaculum mobile* [1]. The genus, described for a thermophilic, citrate-fermenting anaerobe with *A. thermoterrenum* as the type species [2], was emended twice [1,3]. The third species is the recently described *A. hydrogeniformans*, forming H_2_ and acetate from glucose [3] while the other two species form CO_2_. *A. mobile* was isolated from a 10^−7^ dilution of an oleic acid-degrading consortium, originating from the sludge of a wool-scouring wastewater. Enrichment was performed in BCYT medium (basal BC medium [4] with tryptone and yeast extract), supplemented with crotonate [1]. Here we present a summary classification and a set of features for *A. mobile* NGA^T^ together with the description of the complete genomic sequencing and annotation.

## Classification and features

### 16S rRNA gene sequence analysis

A representative genomic 16S rRNA gene sequence of *A. mobile* NGA^T^ was compared using NCBI BLAST [5,6] under default settings (e.g., considering only the high-scoring segment pairs (HSPs) from the best 250 hits) with the most recent release of the Greengenes database [7] and the relative frequencies of taxa and keywords (reduced to their stem [8]) were determined, weighted by BLAST scores. The most frequently occurring genera were *Anaerobaculum* (81.9%), *Acetomicrobium* (9.9%), *Thermovirga* (4.5%) and *Dethiosulfovibrio* (3.6%) (12 hits in total). Regarding the two hits to sequences from members of the species, the average identity within HSPs was 99.9%, whereas the average coverage by HSPs was 100.0%. Regarding the seven hits to sequences from other members of the genus, the average identity within HSPs was 97.1%, whereas the average coverage by HSPs was 99.1%. Among all other species, the one yielding the highest score was *Acetomicrobium flavidum* (FR733692), which corresponded to an identity of 99.8% and an HSP coverage of 100.0%. (Note that the Greengenes database uses the INSDC (= EMBL/NCBI/DDBJ) annotation, which is not an authoritative source for nomenclature or classification.) The highest-scoring environmental sequence was FN436106 (Greengenes name 'Molekularbiologische Charakterisierung der bakteriellen Populationsdynamik eines thermophil betriebenen Biogasfermenters zur Vergaerung nachwachsender Rohstoffe thermophilic biogas reactor fed renewable biomass clone HAW-R60-B-745d-BC'), which showed an identity of 99.9% and an HSP coverage of 99.9%. The most frequently occurring keywords within the labels of all environmental samples which yielded hits were 'digest' (7.0%), 'anaerob' (4.6%), 'thermophil' (4.3%), 'microbi' (3.4%) and 'mesophil' (3.3%) (238 hits in total). The most frequently occurring keywords within the labels of those environmental samples which yielded hits of a higher score than the highest scoring species were 'thermophil' (12.3%), 'reactor' (8.8%), 'bioga, fed' (7.0%), 'bakteriellen, betriebenen, biogasferment, biomass, charakterisierung, molekularbiologisch, nachwachsend, populationsdynamik, renew, rohstoff, vergaerung' (5.3%) and 'corn, microbi, silag, structur' (1.8%) (4 hits in total).

*A. flavidum* is a species that was originally described without 16S rRNA gene sequencing [9]. The sequence FR733692 was only recently been generated by DSMZ staff in the course of the Living-Tree Project [10], yielding an unexpected placement of the species within the *Synergistetes*. An assessment of whether a contamination or confusion of strains has occurred, or whether the currently available culture deposits are biologically identical to the original description but the classification of *A. flavidum* needs to be revised. This work is currently in progress. (R. Pukall (DSMZ), personal communication).

**Figure 1:**
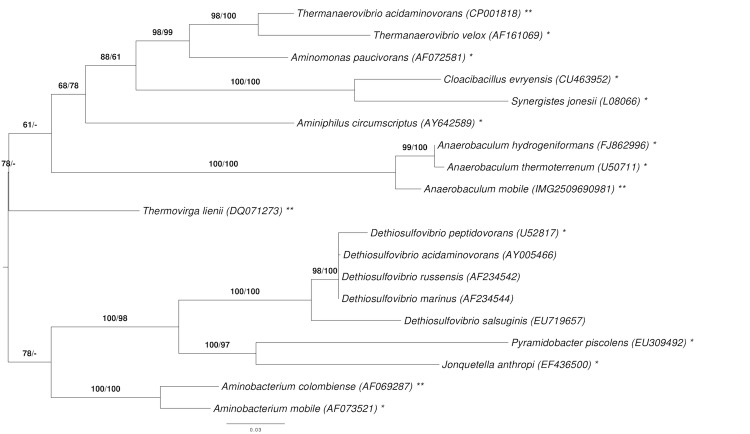
Phylogenetic tree highlighting the position of *A. mobile* relative to the type strains of the other species within the phylum *'Synergistetes'*. The tree was inferred from 1,360 aligned characters [11,12] of the 16S rRNA gene sequence under the maximum likelihood (ML) criterion [13]. Rooting was done initially using the midpoint method [14] and then checked for its agreement with the current classification (Table 1). The branches are scaled in terms of the expected number of substitutions per site. Numbers adjacent to the branches are support values from 1,000 ML bootstrap replicates [15] (left) and from 1,000 maximum-parsimony bootstrap replicates [16] (right) if larger than 60%. Lineages with type strain genome sequencing projects registered in GOLD [17] are labeled with one asterisk, those also listed as 'Complete and Published' with two asterisks [18-20]; the non-contiguous finished draft genomes of *Aminomonas paucivorans* [21] and *Dethiosulfovibrio peptidovorans* [22] lack the second asterisk.

Figure 1 shows the phylogenetic neighborhood of *A. mobile* in a 16S rRNA gene based tree. The sequences of the two identical 16S rRNA gene copies in the genome differ by one nucleotide from the previously published 16S rRNA gene sequence (AJ243189).

**Table 1:** Classification and general features of *A. mobile* NGA^T^ according to the MIGS recommendations [23].

**MIGS ID**	**Property**	**Term**	**Evidence code**
		Domain *Bacteria*	TAS [24]
		Phylum *Synergistetes*	TAS [25]
		Class *Synergistia*	TAS [25]
	Current classification	Order *Synergistales*	TAS [25]
		Family *Synergistaceae*	TAS [25]
		Genus *Anaerobaculum*	TAS [1,3,26]
		Species*Anaerobaculum mobile*	TAS [1]
MIGS-7	Subspecific genetic lineage (strain)	NGA^T^	TAS [1]
MIGS-12	Reference for biomaterial	Menes and Muxí 2002	TAS [1]
	Gram stain	Gram-negative	TAS [1]
	Cell shape	rod-shaped	TAS [1]
	Motility	motile	TAS [1]
	Sporulation	non-sporulating	TAS [1]
	Temperature range	35-65°C	TAS [1]
	Optimum temperature	55-60°C	TAS [1]
	Salinity	optimum growth at 0.8%	TAS [1]
MIGS-22	Relationship to oxygen	obligate anaerobe	TAS [1]
	Carbon source	organic acids and carbohydrates	NAS
	Energy metabolism	chemoorganotroph	TAS [1]
MIGS-6	Habitat	wastewater	TAS [1]
MIGS-6.2	pH	optimum 6.6 - 7.3	TAS 19]
MIGS-15	Biotic relationship	free living	TAS [1]
MIGS-14	Known pathogenicity	none	TAS [1]
MIGS-16	Specific host	not reported	
MIGS-14	Biosafety level	1	TAS [27]
MIGS-19	Trophic level	not reported	
MIGS-23.1	Isolation	wool-scouring wastewater treatment lagoon	TAS [1]
MIGS-4	Geographic location	Trinidad, Uruguay	TAS [1]
MIGS-5	Time of sample collection	November 1998 or earlier	NAS
MIGS-4.1	Latitude	-33.506	TAS [1]
MIGS-4.2	Longitude	-56.889	TAS [1]
MIGS-4.3	Depth	not reported	
MIGS-4.4	Altitude	not reported	

Evidence codes - TAS: Traceable Author Statement (i.e., a direct report exists in the literature); NAS: Non-traceable Author Statement (i.e., not directly observed for the living, isolated sample, but based on a generally accepted property for the species, or anecdotal evidence). Evidence codes are from of the Gene Ontology project [28].

### Morphology and physiology

**Figure 2:**
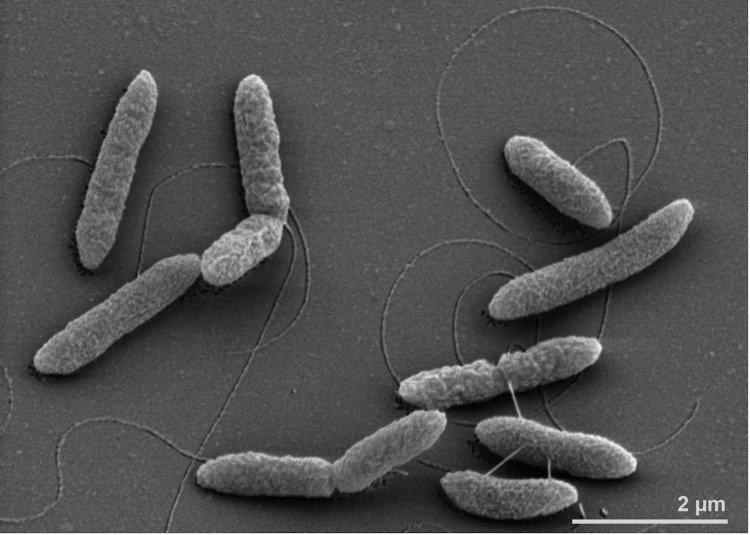
Scanning electron micrograph of *A. mobile* NGA^T^

The Gram-negative rod-shaped cells of *A. mobile* are straight (0.5-1.0 × 2.0-4.0 µm; Figure 2), occurring singly or, predominantly in the exponential growth phase, in pairs. Longer cells (4.0-8.0 µm) are observed in older cultures. Spores or sheath formation were never observed. Cells are moderately thermophilic; motile by means of a single flagellum inserted in the lateral region of the cell. On BCTC agar, colonies (1.0-1.5 mm in diameter) are white, lens shaped and with entire margins. Temperature and pH range for growth is between 35-65°C (optimum between 55 and 60°C) and between 5.4 and 8.7 (optimum between 6.6 and 7.3), respectively. Growth occurs at NaCl concentrations of up to 1.5%, with an optimum of 0.8%.

Cells are strictly anaerobic, fermenting glucose, organic acids (pyruvate, tartrate and malate) and glycerol to acetate, H_2_ and, presumably, CO_2_ (the authors of [1] assumed that 2 mol CO_2_ was produced per mol glucose, tartrate and malate degraded, and 1 mol CO_2_ was produced per mol pyruvate and glycerol degraded). Gluconate, L-malate, glycerol, tryptone, L-arginine, L-leucin, L-phenylalanine and starch are utilized [3]. No growth occurs on citrate, 2-oxoglutarate, glutamate, mannose, pectin, lactose, xylose, galactose, maltose, sucrose, rhamnose, raffinose, malonate, lactate, succinate, xylan, dextrin, inulin, melibiose, adonitol, cellobiose, arabinose, polygalacturonate, cellulose, gelatin, butyrate or oleate [1], nor lactate, maltose, malonate, mannose, inositol and inulin [3].

In co-culture with *Methanothermobacter thermautotrophicus* glucose and malate degradation were enhanced. Crotonate is not fermented but used as an electron acceptor, reducing it to butyrate in the presence of yeast extract and tryptone, glucose, Casamino acids and leucine. Fumarate, acetate, sulfate, sulfite and nitrate are not used as electron acceptors in PY broth. The addition of crotonate, sulfur, cystine and thiosulfate enhances growth from tryptone and yeast extract, Casamino acids and glucose, as indicated by an increase in OD and by the production of reduction compounds (butyrate and sulfide, respectively) [1].

### Chemotaxonomy

The cell wall is of the Gram-negative type as judged by transmission electron micrography [1]. Polar lipid composition includes diphosphatidylglycerol, phosphatidylglycerol, phosphatidylethanolamine, three unknown phospholipids and four unknown aminophospholipids [3]. Major fatty acids (>20%) are *iso*-C_15:0_ and *iso*-C_11:0_; *iso*-C_13:0 3-OH_ is found in smaller amounts (<10%) [3]. The DNA G+C content was previously reported with 51.5 mol% [1].

## Genome sequencing and annotation

### Genome project history

This organism was selected for sequencing on the basis of its phylogenetic position [29], and is part of the *** G****enomic* ***E****ncyclopedia of* ***Bacteria**** and* ***Archaea***** project [30]. The genome project is deposited in the Genomes On Line Database [17] and the complete genome sequence is deposited in GenBank. Sequencing, finishing and annotation were performed by the DOE Joint Genome Institute (JGI) using state of the art sequencing technology [31]. A summary of the project information is shown in Table 2.

**Table 2:** Genome sequencing project information

**MIGS ID**	**Property**	**Term**
MIGS-31	Finishing quality	Finished
MIGS-28	Libraries used	Three genomic libraries: one 454 pyrosequence standard library, one 454 PE libraries (11.0 kb insert size), one Illumina library
MIGS-29	Sequencing platforms	Illumina GAii, 454 GS FLX Titanium
MIGS-31.2	Sequencing coverage	1,109.0 × Illumina; 38.3 × pyrosequence
MIGS-30	Assemblers	Newbler version 2.3-PreRelease-6/30/2009, Velvet version 1.0.13, phrap version SPS - 4.24
MIGS-32	Gene calling method	Prodigal 1.4, GenePRIMP
	INSDC ID	CP003198
	GenBank Date of Release	June 14, 2012
	GOLD ID	Gc02248
	NCBI project ID	53351
	Database: IMG	2509601011
MIGS-13	Source material identifier	DSM 13181
	Project relevance	Tree of Life, GEBA

### Growth conditions and DNA isolation

*A mobile* strain NGA^T^, DSM 13181, was grown anaerobically in DSMZ medium 104b (PYX, medium) at 55°C. DNA was isolated from 1-1.5 g of cell paste using a Jetflex Genomic DNA Purification Kit (GENOMED 600100) and following the standard protocol as recommended by the manufacturer with modification (an additional 10 µl proteinase K digestion for cell lysis was added; 40 min incubation at 58°C). DNA is available through the DNA Bank Network [32].

### Genome sequencing and assembly

The genome was sequenced using a combination of Illumina and 454 sequencing platforms. All general aspects of library construction and sequencing can be found at the JGI website [33]. Pyrosequencing reads were assembled using the Newbler assembler (Roche). The initial Newbler assembly, consisting of 31 contigs in one scaffold, was converted into a phrap [34] assembly by making fake reads from the consensus, to collect the read pairs in the 454 paired end library. Illumina GAii sequencing data (2,649.5 Mb) was assembled with Velvet [35] and the consensus sequences were shredded into 1.5 kb overlapped fake reads and assembled together with the 454 data. The 454 draft assembly was based on 113.8 Mb 454 draft data and all of the 454 paired end data. Newbler parameters are -consed -a 50 -l 350 -g -m -ml 20. The Phred/Phrap/Consed software package [34] was used for sequence assembly and quality assessment in the subsequent finishing process. After the shotgun stage, reads were assembled with parallel phrap (High Performance Software, LLC). Possible mis-assemblies were corrected with gapResolution [33], Dupfinisher [36], or sequencing cloned bridging PCR fragments with subcloning. Gaps between contigs were closed by editing in Consed, by PCR and by Bubble PCR primer walks (J.-F. Chang, unpublished). A total of 68 additional reactions and 3 shatter libraries were necessary to close gaps and to raise the quality of the finished sequence. Illumina reads were also used to correct potential base errors and increase consensus quality using a software Polisher developed at JGI [37]. The error rate of the completed genome sequence is less than 1 in 100,000. Together, the combination of the Illumina and 454 sequencing platforms provided 1147.3 × coverage of the genome. The final assembly contained 237,629 pyrosequence and 32,103,764 Illumina reads.

### Genome annotation

Genes were identified using Prodigal [38] as part of the DOE-JGI genome annotation pipeline [39], followed by a round of manual curation using the JGI GenePRIMP pipeline [40]. The predicted CDSs were translated and used to search the National Center for Biotechnology Information (NCBI) non-redundant database, UniProt, TIGR-Fam, Pfam, PRIAM, KEGG, COG, and InterPro databases. Additional gene prediction analysis and functional annotation was performed within the Integrated Microbial Genomes - Expert Review (IMG-ER) platform [41].

***Genome properties***

**Figure 3:**
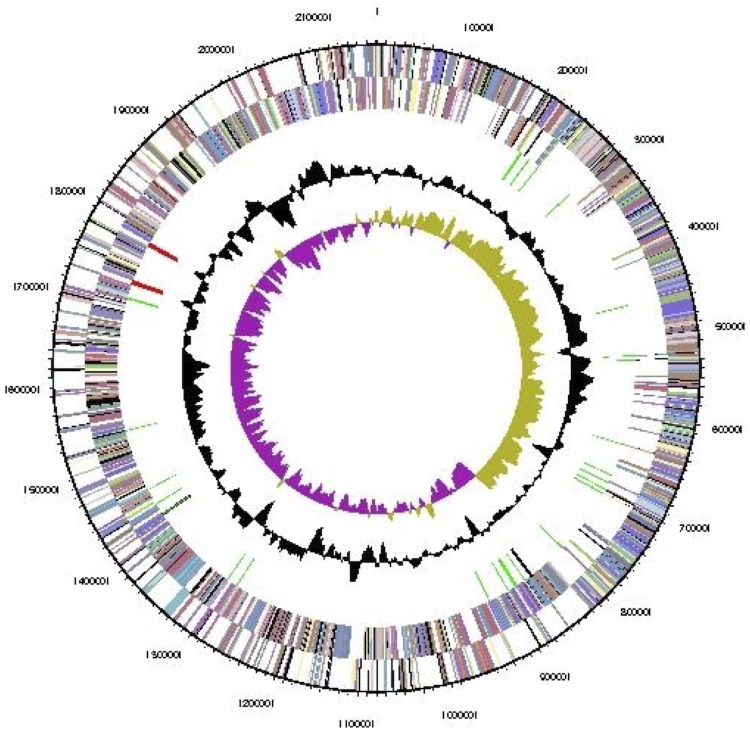
Graphical map of the chromosome. From outside to the center: Genes on forward strand (color by COG categories), Genes on reverse strand (color by COG categories), RNA genes (tRNAs green, rRNAs red, other RNAs black), GC content, GC skew (purple/olive).

The genome statistics are provided in Table 3 and Figure 3. The genome consists of one chromosome with a total length of 2,160,700 bp and a G+C content of 48.0%. Of the 2,109 genes predicted, 2,053 were protein-coding genes, and 56 RNAs; 34 pseudogenes were also identified. The majority of the protein-coding genes (85.7%) were assigned a putative function while the remaining ones were annotated as hypothetical proteins. The distribution of genes into COGs functional categories is presented in Table 4.

**Table 3:** Genome Statistics

**Attribute**	**Number**	**% of Total**
Genome size (bp)	2,160,700	100.00
DNA coding region (bp)	1,982,774	91.77
DNA G+C content (bp)	1,036,446	47.97
Number of replicons	1	
Extrachromosomal elements	0	
Total genes	2,109	100.00
RNA genes	56	2.66
rRNA operons	2	
tRNA genes	48	2.28
Protein-coding genes	2,053	97.34
Pseudo genes	34	1.61
Genes with function prediction	1,808	85.73
Genes in paralog clusters	881	41.77
Genes assigned to COGs	1,796	85.16
Genes assigned Pfam domains	1,809	85.78
Genes with signal peptides	276	13.09
Genes with transmembrane helices	487	23.09
CRISPR repeats	3	

**Table 4:** Number of genes associated with the general COG functional categories

**Code**	**Value**	**%age**	**Description**
J	149	7.51	Translation, ribosomal structure and biogenesis
A	0	0.00	RNA processing and modification
K	86	4.34	Transcription
L	102	5.14	Replication, recombination and repair
B	0	0.00	Chromatin structure and dynamics
D	31	1.56	Cell cycle control, cell division, chromosome partitioning
Y	0	0.00	Nuclear structure
V	16	0.81	Defense mechanisms
T	56	2.82	Signal transduction mechanisms
M	117	5.90	Cell wall/membrane/envelope biogenesis
N	63	3.18	Cell motility
Z	0	0.00	Cytoskeleton
W	0	0.00	Extracellular structures
U	41	2.07	Intracellular trafficking, secretion, and vesicular transport
O	61	3.08	Posttranslational modification, protein turnover, chaperones
C	176	8.88	Energy production and conversion
G	131	6.61	Carbohydrate transport and metabolism
E	263	13.26	Amino acid transport and metabolism
F	69	3.48	Nucleotide transport and metabolism
H	85	4.29	Coenzyme transport and metabolism
I	34	1.71	Lipid transport and metabolism
P	92	4.64	Inorganic ion transport and metabolism
Q	36	1.82	Secondary metabolites biosynthesis, transport and catabolism
R	218	10.99	General function prediction only
S	157	7.92	Function unknown
-	313	14.84	Not in COGs
